# Challenges of Emergency Low-Skill Fibreoptic Intubation in Airway Obstruction by a Neck Haematoma After the Sistrunk Procedure

**DOI:** 10.7759/cureus.65937

**Published:** 2024-08-01

**Authors:** Ofelia Lim Yiqi, Angie Phui-Sze Au-Yong

**Affiliations:** 1 Department of Anaesthesiology, Singapore General Hospital, Singapore, SGP; 2 Department of Anaesthesiology, Sengkang General Hospital, Singapore, SGP

**Keywords:** sistrunk procedure, laryngeal mask airway (lma), neck haematoma, low-skill fibreoptic intubation, difficult airway management

## Abstract

Anterior neck haematoma is a rare but potentially fatal emergency due to airway obstruction after a surgical intervention of the neck. Complete airway obstruction can be rapid and deteriorate exponentially. In a patient with a previously normal laryngeal view during his Sistrunk procedure, we describe the challenges and considerations faced in his second surgery for the evacuation of neck haematoma, which involved an unanticipated difficult airway after rapid sequence induction, necessitating rescue measures using low-skill fibreoptic intubation (LSFOI).

## Introduction

Fibreoptic intubation (FOI) is the gold standard [1] in the management of difficult airway. The intubating fibreoptic bronchoscope (FOB) was first described in 1967 by Peter Murphy [2], and there are various approaches to using it. Low-skill FOI (LSFOI) is a method done through a supraglottic airway device (SAD); it can be done via either the direct or the indirect approach (with an Aintree catheter). The SAD allows ventilation during and between intubation attempts, acting as a conduit to guide the bronchoscope to the glottis after exiting the bowl of the SAD. The latter two functions make it "low skill" [3]. It has a high success rate and is recommended as a Plan B in an unanticipated difficult intubation in the 2015 Difficult Airway Society (DAS) guidelines after a maximum of 3+1 attempts at tracheal intubation [4].

The Sistrunk procedure entails the removal of the thyroglossal duct remnants with a portion of the hyoid bone. Given the anatomic location, thyroglossal duct cyst removal is associated with a risk of neck haematoma which can lead to airway obstruction. Impaired venous and lymphatic drainage causes oedema and distortion of glottic and supraglottic structures [5]. Thus, the ensuing management of the upper airway becomes more challenging. Relief of the neck haematoma may prevent existing oedema from worsening without improving airway patency. Options for airway rescue after the Sistrunk procedure also differ from a post-thyroidectomy situation because opening all layers of a post-thyroidectomy wound exposes the trachea, enabling rapid access for emergency tracheostomy.

The challenges of LSFOI can occur due to difficulties with the manipulation of the SAD, the fibreoptic scope, or the Aintree intubation catheter (AIC) [3]. SAD-related difficulties can occur when the curvature of the SAD is too acute or when there is a suboptimal or obscured glottic view resulting in difficulty passing the tracheal tube through the SAD. FOB-related difficulties can occur when there is dislodgement of the SAD on the removal of the FOB or inadvertent FOB advancement into the oesophagus. AIC-related difficulties result from difficulty railroading the tracheal tube over the AIC or dislodgement or kinking of the AIC. 

We describe the challenges and considerations faced in the second surgery for the evacuation of neck haematoma, which involved an unanticipated difficult airway after rapid sequence induction, necessitating rescue measures using LSFOI.

## Case presentation

A 64-year-old male patient, 162 cm and 85 kg (BMI 32.4), with a past medical history significant for obstructive sleep apnoea (OSA) for which he previously had an inferior turbinoplasty and uvulopalatopharyngoplasty in 2014, underwent an elective Sistrunk surgery for a large (4.6×4.2×1.7 cm) infrahyoid complex thyroglossal cyst (located along the midline of the anterior neck) and excision of a 2 cm right neck lipoma. Preoperative airway examination was unremarkable: he was a Mallampati 1 (albeit the uvula was absent given his previous surgery), thyromental distance was adequate, and neck range of motion was full. After standard American Society of Anesthesiologists (ASA) monitoring was applied, he was induced with IV fentanyl 75 mcg, IV lignocaine 30 mg, IV propofol 120 mg, and IV atracurium 40 mg. He had a Cormack-Lehane grade 1 larynx on direct laryngoscopy and was intubated uneventfully with a size 8 endotracheal tube (ETT). Postoperatively, the patient was extubated and discharged to the general ward.

However, five hours later, he started to develop neck swelling, dyspnoea, and dysphagia. The surgical team diagnosed a large neck haematoma and immediately released the neck sutures at the bedside in the ward. Copious clots were evacuated, but due to persistent brisk bleeding, he was urgently transferred to the emergency operating theatre (OT) for the evacuation of neck haematoma and haemostasis. Estimated blood loss was about 300 ml prior to OT. On arrival, he was haemodynamically stable with a SpO2 of 98% on 5 L/min via nasal prong O2. There was no stridor. As such, given that he had previously been a grade 1 larynx on direct laryngoscopy and there were no signs of impending airway obstruction after the release of the neck haematoma, the decision was made for asleep intubation with a videolaryngoscope.

The patient was induced with IV midazolam 1 mg, IV fentanyl 75 mcg, IV propofol 150 mg, and IV succinylcholine 100 mg. Videolaryngoscopy was done by the most senior anaesthetist with McGrath MAC 4 blade, revealing a poor laryngeal view where the epiglottis could not be visualised. There was substantial swelling in the base of the tongue which made insertion of the laryngoscope blade difficult. A second attempt was performed with a McGrath X blade after optimising patient position; the epiglottis could be visualised, but even a #6 ETT with a rigid stylet could not be passed through the vocal cords due to extensive laryngeal oedema and anterior positioning of the larynx. A third attempt was performed with a McGrath X blade and a #6 ETT loaded over a 3.8 mm disposable Ambu FOB, but the bronchoscope could not be directed through the vocal cords, likely due to limited space for manoeuvring from surrounding tissue oedema and distorted anatomy.

Anaesthetic depth was maintained throughout with a further IV 1 mg of midazolam and target-controlled infusion (TCI) propofol at 2.5 mcg/ml. In between intubation attempts, adequate bag-mask ventilation with oral airway was performed. The lowest SpO2 recorded was 85% transiently after the first intubation attempt; otherwise, SpO2 was always above 95%. Given that there were already three attempts at intubation, consideration was given to the risks of worsening laryngeal oedema with a fourth airway manipulation versus proceeding to a surgical airway. The option of aborting intubation and waking the patient up was deemed not feasible because the evacuation of clots and haemostasis still had to be achieved. As bag-mask ventilation was still adequate at maintaining oxygenation, the decision was made to make one last intubation attempt while the surgeon prepared for surgical tracheostomy.

Before the last intubation attempt, IV 50 mg rocuronium was administered as the succinylcholine given earlier would likely have worn off. LSFOI was attempted successfully through the size 4 Ambu AuraGain laryngeal mask airway (LMA) and a #6 ETT railroaded over the Ambu 3.8 mm scope [6]. Once the airway was secured, the operation proceeded uneventfully, and haemostasis of the bleeding areas was achieved (there was brisk bleeding from the cut suprahyoid muscle edge on the left, as well as general ooze across the rest of the wound bed). IV dexamethasone 8 mg was given to reduce airway swelling.

Postoperatively, the patient was kept intubated and planned for admission to the intensive care unit (ICU) in view of significant airway oedema from the neck haematoma and multiple intubation attempts. Before transfer to the ICU, the decision was made to remove the LMA due to concerns of mucosal pressure necrosis if it was left in situ. A 14F 83 cm Cook airway exchange catheter [7] was inserted through the ETT prior to LMA removal, and the ability to ventilate through the airway exchanger was confirmed prior to removing the LMA. However, on the removal of the LMA, the ETT and airway exchanger were inadvertently dislodged. Reintubation was attempted via LSFOI with the Aintree catheter mounted onto the Ambu scope, but the laryngeal inlet could not be visualised. Videolaryngoscopy with McGrath X blade was attempted; the laryngeal grade had improved to a grade 2a, although the laryngeal aperture remained small from the surrounding oedema. The patient was intubated successfully with a #6 ETT.

## Discussion

Challenges of LSFOI

This report discusses the challenges of emergency LSFOI during impending airway compromise from a postoperative neck wound haematoma after a Sistrunk procedure. This patient had a large (4.6×4.2×1.7 cm) complex thyroglossal cyst interdigitating with overlying strap muscles and an obvious tract leading into the tongue base. The Sistrunk procedure involves surgical resection of the thyroglossal duct to the base of the tongue in addition to excision of the cyst and the middle segment of the hyoid bone. Although the patient had an easy intubation for the first operation, the neck haematoma likely resulted in the significant base of tongue and laryngeal swelling due to the impairment of venous drainage, persisting even after sutures had been released and contributed by ongoing bleeding within the neck. The degree of oedema does not necessarily correlate with the severity of external swelling and may not resolve immediately upon clot evacuation [8].

Consideration was given to whether intubation should be done awake or asleep. Factors in favour of asleep intubation included the patient's inability to cooperate as he complained of dyspnoea and dysphagia, and there was brisk bleeding from the neck wound which limited the time available to achieve adequate airway topicalisation. There was also a concern of a possible "cork in the bottle" phenomenon [9], where a narrowed laryngeal aperture could be completely occluded by the scope. Hence, in anticipation of a difficult airway, the decision was made for an asleep intubation with a smaller-sized ETT and a videolaryngoscope instead of direct laryngoscopy. Standby airway adjuncts were prepared, and the ENT surgeon was present in OT and ready for tracheostomy. 

We were cognisant that failed intubation attempts could have traumatised the airway; however, while there was airway oedema from the extrinsic haematoma compression, there was no internal airway bleeding. There was an intense discussion with the primary ENT consultant surgeon (who was present at both the first and second surgeries) to head straight for a tracheostomy; however, this was rejected as the initial solution as she felt that the external bleeding could be repaired without having to do an invasive airway procedure. Hence, the consensus was to prepare for tracheostomy only as the last resort; she was scrubbed up and ready next to the patient during the entire anaesthetic process.

While the laryngeal grade was expected to have deteriorated from the initial surgery, the base of tongue swelling was unexpected. However, this was later apparent when the surgical notes were reviewed and there was documentation of tract excision from the base of the tongue. Subsequently after three unsuccessful attempts, an Ambu fibreoptic scope was attempted as a flexible stylet with a hyperangulated McGrath X blade to enable better visualisation and ETT advancement towards the swollen glottic opening. When this was unsuccessful, the decision was made for LSFOI via an Ambu LMA. As the Ambu AuraGain is a preformed LMA, it allowed for the smooth conduit of the fibreoptic scope without impedance from the swollen base of the tongue and facilitated the railroading of the #6 ETT through the shaft of the LMA. LSFOI can therefore be used as a last-resort rescue method as the LMA can be used to keep the patient ventilated and oxygenated in between intubation attempts.

Studies have shown that a larger distance between a fibreoptic scope and an endotracheal tube results in an increased likelihood of difficulty in sliding the tube down the fibreoptic scope (direct LSFOI) [10]. Minimising this gap alleviates the difficulty of advancing the tube. This can be done by either using a thicker fibreoptic scope with a narrower-diameter tracheal tube or using a gap filler such as an Aintree catheter mounted onto the fibreoptic scope (indirect LSFOI approach) [10].

Should the LMA be removed after LSFOI

Postoperatively, LMA removal was attempted due to concerns of mucosal pressure necrosis and ICU nursing unfamiliarity with LMA. Additionally, LMA is not recommended for elective ventilation in the ICU. Despite the use of a long 83 cm Cook airway exchange catheter [7] as a bougie (compared to the shorter 56 cm Aintree intubation catheter), it was still inadvertently dislodged when attempting the removal of the LMA. Nevertheless, it has been recommended that the maximum depth a bougie or airway exchange catheter should be inserted is 26 cm to avoid damage from entry into the distal tracheobronchial tree [11]. It has also been suggested that the LMA should not be removed after its use in LSFOI, as it provides an alternative airway in situ [12].

Numerous challenges have been described when an ETT is inserted through an LMA, especially upon LMA removal after a successful intubation [13]. Of note, the length of the LMA's airway tube can be similar to the ETT, rendering it challenging to maintain control of the ETT while removing the LMA. The nominal length of the internal ventilatory pathway for a size 4 LMA Ambu AuraGain is 17.5+/-1.1 cm [6]. A bronchoscopic study by Asai et al. showed that the mean distance between the grille of the LMA and the vocal cords was 3.6 cm (range 2.5-4.7 cm) in males and 3.1 cm (range 2.0-4.2 cm) in females [14]. In a size 6 ETT, the distance between the upper edge of the cuff and the tip of the tracheal tube is 5 cm. This suggests firstly that the cuff of an uncut size 6 ETT (length 28 cm) may often lie between the vocal cords even if the tube is fully inserted through the LMA and secondly that the margin for manoeuvring the LMA over the ETT without dislodging the latter is small.

Techniques that minimise the possibility of accidental ETT extubation during LMA removal have been invented and reported in the literature. These include using a long ETT, a dual ETT manoeuvre (a second, proximal ETT is used to hold the first and more distal ETT in the trachea while removing the LMA), truncating or splitting the LMA shaft, or leaving the LMA in situ for the duration of surgery [13]. Literature is sparse about whether an LMA should be removed post-LSFOI, and there is no common consensus as to the appropriate method of removal. While the conjoined Association of Paediatric Anaesthetists and DAS guidelines recommend leaving the LMA in place after intubation [15], cutting the LMA due to concerns of ETT dislodgement from LMA traction in a difficult paediatric airway at impending risk of obstruction from peritonsillar abscess has also been reported [16].

Extended use of an LMA is generally not recommended as prolonged LMA cuff inflation is believed to result in substantial transmitted mucosal pressures that can potentially exceed the capillary perfusion pressure of the adjacent pharyngeal mucosa and cause mucosal necrosis [17]. The i-gel manufacturers recommend that the i-gel be used for not more than four hours [18]. Its cuff is made of a medical-grade thermoplastic elastomer to create a non-inflatable anatomical seal of the pharyngeal, laryngeal, and perilaryngeal structures while avoiding the compression trauma that can occur with inflatable supraglottic airway devices. It has a narrower tip and forms a seal lower down in the oesophagus which reduces the risk of dysphagia compared with other supraglottic airway devices. There are reports of the i-gel being used for mechanical ventilation in the ICU for 27 hours [19] with no adverse effects. The use of an LMA has also been reported in the ICU setting as an alternative method of weaning ventilatory support post-extubation for up to 16 hours [20]. 

## Conclusions

LSFOI is a useful technique to rescue difficult airways. In a successful LSFOI, consideration should be given to the extubation process. If the patient is anticipated for prolonged intubation in the ICU, a delayed removal of LMA can be done after laryngeal swelling is reduced. If anticipated to be extubated within the next 1-3 days, there is a role for leaving the LMA in situ with a deflated cuff and tracheal tube in situ to reduce the risk of inadvertent airway dislodgement while attempting to remove the LMA.
